# Revolutionized attitude toward recurrent pregnancy loss and recurrent implantation failure based on precision regenerative medicine

**DOI:** 10.1016/j.heliyon.2024.e39584

**Published:** 2024-10-18

**Authors:** Kimia Motlagh Asghari, Tannaz Novinbahador, Amir Mehdizadeh, Mohammadali Zolfaghari, Mehdi Yousefi

**Affiliations:** a Immunology Research Center, Tabriz University of Medical Sciences, Tabriz, Iran; b Department of Biology, Faculty of Natural Sciences, University of Tabriz, Tabriz, Iran; c Department of Clinical Biochemistry, Faculty of Medicine, Tabriz University of Medical Sciences, Tabriz, Iran; d Hematology and Oncology Research Center, Tabriz University of Medical Sciences, Tabriz, Iran

**Keywords:** Recurrent pregnancy loss, Recurrent implantation failure, Regenerative medicine, Precision regenerative medicine, Infertility

## Abstract

Traditional treatment strategies for recurrent pregnancy loss (RPL) and recurrent implantation failure (RIF) often result in limited success, placing significant emotional and financial burdens on couples. However, novel approaches such as diagnostic gene profiling, cell therapy, stem cell-derived exosome therapy, and pharmacogenomics offer promising, personalized treatments. Combining traditional treatments with precision and regenerative medicine may enhance the efficacy of these approaches and improve pregnancy outcomes. This review explores how integrating these strategies can potentially transform the lives of couples experiencing repeated pregnancy loss or implantation failure, providing hope for improved treatment success. Precision and regenerative medicine represent a new frontier for managing RPL and RIF, offering promising solutions.

## Highlights

• Gene profiling, cell therapy, and exosome treatments offer new treatments for recurrent implantation and pregnancy failure.
• Combining traditional methods with precision medicine enhances treatment outcomes.
• Regenerative medicine brings new hope to couples facing these challenges.

## 1 Introduction

Recurrent pregnancy loss (RPL) and recurrent implantation failure (RIF) are complex, multifactorial conditions that can profoundly impact the lives of affected individuals. RPL is defined as two or more consecutive pregnancy losses before 20 weeks of gestation [1]. RIF, conversely, refers to the failure of two or more consecutive in vitro fertilization (IVF) cycles despite using high-quality embryos [2]. These conditions not only affect couples' attempts to achieve a successful pregnancy but may also lead to long-term reproductive and psychological challenges [3,4].

Despite significant advances in medical science, the underlying causes of RPL and RIF remain largely unknown. Genetic and chromosomal abnormalities, uterine defects, hormonal imbalances, immune dysregulation, and environmental factors have all been implicated [[5], [6], [7], [8], [9], [10]]. Current therapeutic options, such as progesterone, aspirin, and intravenous immunoglobulin (IVIG), offer limited efficacy and do not often address the root causes [[10], [11], [12], [13]].

In recent years, however, precision and regenerative medicine have emerged as promising strategies to manage RPL and RIF. Precision medicine aims to personalize healthcare by integrating genetic, environmental, and lifestyle factors. For instance, diagnostic gene profiling helps identify genetic variations associated with these conditions [14]. Regenerative medicine, a broad field encompassing tissue engineering, stem cell therapy, and biomaterials, introduces cell-based treatments and pharmacogenomics for developing personalized therapeutic strategies [15]. These approaches hold the potential to revolutionize the management of RPL and RIF, offering renewed hope for couples facing these reproductive challenges.

This review will comprehensively overview the latest precision and regenerative medicine advancements for managing RPL and RIF. We will explore diagnostic gene profiling, assess the role of cell and exosome therapy, and examine the potential of pharmacogenomics in developing individualized treatment plans. This article aims to provide valuable insights into the future of RPL and RIF treatment by presenting the latest developments.

## 2 Diagnostic gene profiles

Recent evidence underscores the role genetics play in RIF and RPL. Advances in genetic testing and next-generation sequencing (NGS) have opened new opportunities for precision medicine in these fields [16]. Genetic testing for RIF and RPL involves analyzing the patient's and their partner's DNA to identify genetic variants linked to these conditions. These tests can be performed on parental or fetal/placental tissue to determine potential genetic causes. Variants may be inherited or occur spontaneously during embryo development [17]. Various testing methods, such as karyotyping, chromosomal microarray analysis (CMA), fluorescence in situ hybridization (FISH), and NGS, are used to identify these variants [18,19]. Karyotyping and FISH are traditional methods that detect significant chromosomal abnormalities associated with RPL and RIF but may miss minor genetic variants [20]. CMA, a newer test, detects more variants but has limitations in identifying more minor or novel mutations [21,22]. NGS offers the highest resolution and can identify various genetic variants, including single nucleotide polymorphisms (SNPs), that may contribute to RPL and RIF [23,24]. Preimplantation genetic testing for aneuploidy (PGT-A) is helpful during IVF to detect chromosomal abnormalities in embryos before implantation [25,26]. Additionally, the NGS-based PGT-A may be a valuable supplement for RIF management [26,27]. Array comparative genomic hybridization (aCGH) can also identify genetic abnormalities [28]. PGT-A is a crucial component of IVF cycles for patients with RIF, aiming to identify chromosomal abnormalities in embryos before implantation. Genetic testing of embryos allows clinicians to select euploid embryos with a higher likelihood of implantation and subsequent successful pregnancy, thereby improving IVF success rates [25,26]. The integration of NGS technologies in PGT-A enhances the accuracy and efficiency of embryo screening, offering more significant insights into chromosomal integrity and facilitating informed decisions regarding embryo transfer [26,27]. According to a recent systematic review of studies, PGT-A, based on comprehensive chromosomal screening procedures and blastocyst biopsy in RIF/RPL, conclusively optimizes the reproductive outcomes of these patients, particularly those of advanced age [29].

Several genes, such as those related to thrombophilia, have been linked to RPL and RIF. Thrombophilic genes can increase the risk of blood clots, impair placental development, and promote inflammation, contributing to these conditions [30,31]. A study used NGS to evaluate the association of 29 genotypes of 10 coagulation pathway genes with RPL-RIF in 540 female subjects [32]. This study found that genotypes of seven thrombophilia genes, including MTHFR, SERPINE1, MTR, ANXA5, MTRR PROZ, and VEGFA, are associated with inherited thrombophilia risk for RPL-RIF [32]. Factor V G1691A, factor II prothrombin G20210A, factor XIII V34L, factor V H1299R HPA1 a/b(L33P), *β*-fibrinogen −455G > A, PAI-1 4G/5G, have also shown associations with RPL [33]. Ensuring a sufficient blood supply and angiogenesis is essential for several steps in the early phases of human pregnancy. The angiogenesis and vasoconstriction-related genes are also associated with RPL through mechanisms such as good blood supply, oxidative stress, and endothelial dysfunction [34,35]. These genetic associations are vascular endothelial growth factor (VEGF) (−1154G > A) polymorphisms, p53 (codon72), and endothelial nitric oxide synthase (eNOS) (B/A, Glu298Asp) [36]. Mutations in the FOXD1 gene are also related to RIF, intrauterine growth restriction, and preeclampsia through the regulation of angiogenesis and vasoconstriction-related genes, including complement component 3 (C3) and placental growth factor (PlGF) [37,38]. Mutations in the *SYCP3* gene are also associated with RPL [39]. SYCP3 is involved in the complex synaptonemal formation during meiosis and mutation meiosis, and mutations in this gene have been linked to impaired meiotic progression and infertility in humans [40,41]. Various cytokine gene polymorphisms have also been associated with RPL, particularly in genes encoding TNF-a, IL-1, IFN-g, IL-12, IL-18, IL-4, IL-6, and IL-10 [42]. Quintero-Ronderos et al. [43] were the first to review the most relevant studies involving NGS sequencing, describing the variants related to RPL pathogenesis. A recent study using RNA sequencing identified 213 mRNAs and 1485 lncRNAs that are differentially expressed in the endometrium of RIF patients, with many related to immune and inflammatory processes. Five essential genes (TTR, ALB, TF, AFP, and CFTR) and a module of 14 hub genes were highlighted in a protein-protein interaction (PPI) network. The drug ML-193, targeting these hub genes, significantly enhanced endometrial receptivity by increasing endometrial cell proliferation and upregulating the endometrial receptivity marker HOXA10 [44]. A significant development in understanding endometrial receptivity in RIF is using RNA-sequencing-based diagnostics. A recent study evaluated the effect of a personalized embryo transfer (pET) guided by the RNA-seq-based endometrial receptivity test (rsERT) on clinical outcomes in RIF patients [45]. The study, which included 155 patients, showed that those who underwent rsERT-guided pET had significantly higher rates of positive human chorionic gonadotropin (β-hCG) (56.3 % vs. 30.5 %) and clinical pregnancy (43.8 % vs. 24.2 %) compared to those who had standard frozen embryo transfer (FET) without rsERT. The study highlights the clinical potential of rsERT-guided pET in improving pregnancy outcomes in RIF patients, particularly by targeting personalized embryo transfer based on endometrial receptivity status [45].

Understanding the genetic landscape of RPL and RIF enables a tailored approach to treatment, with interventions aimed at addressing specific genetic variants associated with these conditions. The correlation between genetic testing results and treatment outcomes is fundamental in guiding therapeutic decisions and optimizing patient care. Thrombophilia-related genes, such as MTHFR and SERPINE1, predispose individuals to coagulation abnormalities, increasing the risk of thrombotic events that can compromise pregnancy [32]. Identifying these genetic variants through testing informs the use of anticoagulant therapy, including aspirin and low-molecular-weight heparin (LMWH), which can mitigate the risk of thrombosis and improve pregnancy outcomes. Dosage and administration protocols for anticoagulant therapy are determined based on individual patient factors and genetic profiles, aiming to achieve therapeutic efficacy while minimizing adverse effects. Identifying genetic variants associated with RPL and RIF significantly impacts diagnosis, treatment, and family planning. By integrating genetic testing results with treatment strategies, clinicians can devise personalized therapeutic regimens tailored to the unique genetic profiles of patients with RPL and RIF. This individualized approach enhances treatment efficacy, improves pregnancy outcomes, and fosters a more personalized and precision-oriented paradigm in reproductive medicine.

## 3 Cell therapy

Cell therapy is an emerging treatment modality that uses cells to repair or replace damaged tissues and organs [46]. Recently, cell-based therapies have gained attention as potential treatments for various medical conditions, including RPL and RIF [[47], [48], [49], [50]]. The concept behind cell therapy in these conditions is to address immune and inflammatory abnormalities that may create a hostile uterine environment, preventing successful embryo implantation or causing miscarriage [8,47,51,52]. The uterine immune microenvironment plays a crucial role in pregnancy, with immune cells and cytokines as key regulators. Cell therapy aims to restore immune balance and improve the chances of successful implantation [53]. Cell therapy offers novel therapeutic platforms for various clinical diseases [54,55]. While successful examples of cell-based therapies exist, recent molecular and cellular biology advancements have expanded their potential applications. Cell-based treatments efficiently address patients' pathophysiological conditions, offering dynamic, interactive, and personalized treatments. It holds promise for drug delivery, immunotherapy, and regenerative or replacement engineering of tissues [56].

Peripheral Blood Mononuclear cell (PBMC) treatment operates through several mechanisms to improve endometrial receptivity and promote successful embryo implantation. Upon intrauterine administration, PBMCs release cytokines such as IL-1α, IL-1β, and TNF-α, which play crucial roles in modulating the immune response within the uterine environment. These cytokines positively influence endometrial receptivity, facilitating implantation [53,57]. Additionally, PBMCs contribute to regulating immune tolerance, which is essential for preventing embryo rejection by the maternal immune system during implantation [58,59]. Furthermore, PBMCs enhance endometrial receptivity by promoting the attachment and invasion of the embryo into the endometrial stromal tissue [60,61]. This process involves the secretion of various factors, including IL-4, IL-10, and Th-2-related cytokines, which contribute to embryo-maternal cross-talk and induce endometrial differentiation [62]. Moreover, PBMCs stimulate progesterone production by luteal cells, further supporting the establishment and maintenance of pregnancy [62]. Additionally, human chorionic gonadotropin (hCG) has been shown to induce PBMCs to secrete IL-8 and IL-1, critical factors in embryo implantation [62,63]. Furthermore, hCG enhances the in vitro invasive potential of trophoblast cells to the extracellular matrix, one of the crucial steps in embryo implantation [64].

Lymphocyte therapy, particularly regulatory B cells (B10 cells), has inhibited harmful immune responses and improved pregnancy outcomes in animal models [65]. Studies have demonstrated that transferring B10 cells from normal pregnant mice to abortion-prone animals can prevent fetal rejection [65]. This beneficial function may be facilitated through dendritic cells (DCs) that express IL-10 receptors and can modify the behavior of other immune cells [66]. Furthermore, transferring B10 cells leads to a reduction in mature DCs and an increase in CD4, FOXP3, and Treg levels [67]. These B10 cells in the spleen help maintain the migratory DCs in an immature state, potentially contributing to a pregnancy-supportive environment [48,65]. The utilization of B10 cells with anti-inflammatory properties provides a novel approach to the treatment of immune-related spontaneous abortion.

B cells play a role in producing asymmetric IgG-type antibodies, which are believed to safeguard the semi-allograft fetus by blocking placental antigens and suppressing maternal immune attacks by NK cells and cytotoxic lymphocytes [68]. Hormone regulation is thought to influence the secretion of these asymmetric antibodies [69]. Research has shown that hCG can increase the production of IL-10 and instigate the synthesis of asymmetric antibodies within B cells [70]. Lymphocyte immunotherapy has also been observed to cause alterations in lymphocyte subsets and their functions, which may have potential benefits for maintaining pregnancy and serve as predictive markers for subsequent abortions [71]. High-dose IVIG administration with minimum risk is beneficial for patients experiencing RIF [[72], [73], [74], [75], [76]]. T cell subsets, including Th1, Th2, Th17, and Tregs, are also closely related to feto-maternal immune tolerance during pregnancy and are associated with developing RPL and RIF [71,[77], [78], [79]]. Th1 cells, through pro-inflammatory cytokines such as IL-2 and INF-γ, enhance the cytotoxic function of NK cells, thereby impeding embryo implantation, while Th2 cells, through anti-inflammatory cytokines like IL-4 and IL-10, protect the embryo by suppressing Th1 responses [80]. CD4^+^CD25+FOXP3+ Tregs are crucial for inducing and maintaining tolerance to the semi-allograft fetus [81].

Immunotherapy involving lymphocytes from the father or a third party has demonstrated efficacy in treating unexplained recurrent spontaneous abortion by preserving the balance between Th1 and Th2 cells and promoting Tregs [82]. This treatment strategy has the potential to improve the immune environment for embryo implantation by producing blocking antibodies, disrupting natural killer (NK) cell activity, and maintaining a balance between Th1/Th2 cells and regulatory T cells (Tregs) [83,84]. In a study conducted by Aslanian et al. [85], 200 RPL patients and 200 healthy controls were included to assess the effectiveness of lymphocyte immunotherapy in altering immunological responses in patients with RPL. The study's results revealed that following lymphocyte therapy, there was a significant modification in immune responses: the frequency of Th17 lymphocytes and NK cell cytotoxicity decreased, while the frequency of Treg lymphocytes increased. These changes were also observed at the transcriptional level, as evidenced by RORγt and FoxP3 mRNA expression shifts. Furthermore, lymphocyte therapy was associated with reduced expression of miR-326a and miR-155 and increased expression of miR-146a and miR-10a in RPL patients [85].

A study by Menguan Liu et al. [86] demonstrated that administering low-dose lymphocyte immunotherapy before and during pregnancy significantly influences the immune profile. The therapy effectively reduced the levels of Th1 cells and increased the levels of Th2 cells, Tregs, and the concentration of transforming growth factor-beta 1 (TGF-β1) in the serum following lymphocyte immunotherapy [86]. TGF-β1 plays a crucial role in promoting the differentiation of Tregs from CD4^+^CD25+T cells and inhibiting the activity of IFN-γ and TNF-α [86,87]. This ultimately leads to the expression of FOXP3, a transcription factor associated with Tregs, contributing to a balanced immune response and creating a favorable environment for a successful pregnancy [86,87]. Lymphocyte immunotherapy has been applied worldwide, alone or in combination, for patients with recurrent miscarriages. Research has indicated higher rates of successful pregnancies among women with recurrent miscarriages who underwent paternal lymphocyte immunotherapy compared to control groups [88]. However, some research has not shown significant effects of immunotherapy over placebo, and concerns regarding potential side effects have been raised [[89], [90], [91]].

Stem cell therapy, explicitly involving Mesenchymal Stem Cells (MSCs), holds therapeutic potential in regulating the production of Th1/Th2 cytokines, protecting the fetus in mice prone to abortion, and treating conditions like premature ovarian failure and endometriosis in animal models [92,93]. MSCs can be derived from various sources, such as umbilical cord blood and adipose tissue, and possess regenerative and immunomodulatory properties. These cells have unique characteristics, including the ability to differentiate into different cell types and self-renewal capability [94,95]. They also secrete trophic factors, including growth factors and cytokines such as VEGF, stromal cell-derived factor-1, fibroblast growth factor, transforming growth factor-β (TGF-β), and IL1 receptor antagonist [96,97]. The mechanism of MSCs involves suppressing inflammatory responses mediated by systemic and decidua T-cells, converting pro-inflammatory macrophages (M1) into an anti-inflammatory phenotype (M2) in the decidua through the interaction between CD200 on MSCs and CD200R on M1. This interaction enhances the production of TNF-stimulated gene-6 (TSG-6) by MSCs, resulting in immunosuppression and a decrease in fetal loss [98]. Another study demonstrated that MSCs decreased the abortion rate in mice prone to abortion. This effect was achieved through the downregulation of Th1 cytokines, the induction of Th2 cytokines, and a decrease in lymphocyte proliferation response to paternal antigens [99]. Another study demonstrated that MSC therapy reduced the abortion rate and improved pregnancy outcomes in mice through the upregulation of IL-10 and TGF-β and downregulation of TNF-α, as well as the expression of IFN-γ mRNA [100]. Endometrial MSCs, known as "endometrial stem cells," have been identified and may have potential applications in treating endometriosis and RIF [101]. In a recent experimental study, the impact of endometrial MSCs on endometrial changes in women with thinned endometrium and RIF was evaluated [101]. The results showed a significant increase in post-treatment endometrial thickness values. Endometrial flow cytometry revealed normalized variables post-treatment. Histopathology also showed substantial improvement. Clinical outcomes following endometrial MSC treatment included a clinical pregnancy rate of 79.31 %, a live birth delivery rate per embryo transfer of 45.45 %, and an ongoing pregnancy rate of 24.14 %. These findings suggest that endometrial MSC inoculation effectively enhances endometrial receptivity and improves clinical pregnancy and live birth rates in RIF patients [101].

The inner mass of blastocysts contains pluripotent stem cells called embryonic stem cells (ESCs) [102]. Although they can differentiate into different kinds of cells, they cannot differentiate into placenta cells. Human ESCs have shown potential in IVF embryo establishment, but their allogeneic nature and ethical/technical limitations pose challenges [103]. Other types of pluripotent stem cells, such as induced pluripotent stem cells (iPSC) and ESCs derived through somatic cell nuclear transfer, offer alternative options for regenerative purposes. Human amniotic epithelial cells (hAECs) derived from the amniotic membrane have shown differentiation potential into various cell types and immunomodulatory effects on immune cells [104]. They can suppress the proliferation and activation of B cells, macrophages, and T cells and alter the cytokine profile [105]. hAECs have demonstrated the ability to inhibit the division of naive CD4^+^ T cells and decrease the production of pro-inflammatory cytokines. Decidual Stromal Cells (DSCs) also play a role in maintaining and developing pregnancy and inducing maternal-fetal tolerance. They have been linked to mesenchymal stromal/stem cells and have shown potential in reducing the resorption/implantation ratio and regulating immune-mediated diseases [106] (Table 1).

**Table 1:** Overview of Cell therapy approaches for RPL and RIF.

Cell Type	Mechanism of Action	Clinical Studies/Findings
PBMCs	Induce cytokine production (IL-1α, IL-1β, TNF-α), enhance receptivity	- Intrauterine PBMC application promotes embryo implantation - PBMC co-culture with luteal cells enhances cytokine production, promoting embryo-maternal cross-talk - Induction of progesterone production by luteal cells
hCG-activated PBMCs	Increase Th2/Th1 cytokine ratio during pregnancy	- Induce secretion of cytokines in PBMC - Enhance the invasive potential of trophoblast cells
B10 cells	Produce IL-10, inhibit harmful immune responses	- Transfer of B10 cells prevents fetal rejection
Lymphocyte Immunotherapy	Balance between Th1 and Th2 cells, promote Tregs	- Reduction of Th1 cells and increase in Th2 cells, Tregs with low-dose lymphocyte immunotherapy
MSCs	Regulate Th1/Th2 cytokines, suppress inflammatory responses	- Reduction in abortion rate, upregulation of IL-10 and TGF-β
		- Potential applications in treating endometriosis and RIF

abbreviations: PBMCs: Peripheral Blood Mononuclear Cells, hCG: Human Chorionic Gonadotropin, B10 cells: Regulatory B Cells, NK cells: Natural Killer Cells, Th1/Th2 cells: T-helper 1/T-helper 2 cells, Tregs: Regulatory T Cells, IVIG: Intravenous Immunoglobulin, PRP: Platelet-Rich Plasma, MSCs: Mesenchymal Stem Cells, IL: Interleukin, TNF-α: Tumor Necrosis Factor-alpha, IFN-γ: Interferon-gamma, TGF-β: Transforming Growth Factor-beta.

It is essential to approach stem cell therapy in reproductive medicine cautiously, considering the challenges and potential risks, such as uncontrolled cell proliferation and the potential for malignancy. Despite these concerns, stem cell therapy offers advantages like convenient sampling and abundant sources. Cell therapy has several potential benefits for treating RPL and RIF. These advantages include targeted therapy, regenerative properties, low risk of side effects, and personalized treatment. Cell therapy can provide targeted treatment to address the underlying causes of RPL and RIF, such as immune dysfunction, tissue damage, or genetic abnormalities. Certain types of cells used in cell therapy can promote tissue regeneration and repair. Cell therapy poses a reduced risk of side effects compared to conventional treatments, such as hormone therapy or surgery. Cell therapy can be tailored to the individual needs of the patient, taking into account their medical history, genetic makeup, and other factors. However, there are also several potential disadvantages associated with cell therapy. Cell therapy is a relatively new field of medicine, and there is still limited research on its long-term safety and efficacy. Cell therapy can be expensive and may not be widely available. While the risk of complications is low, there is still a risk of infection, immune rejection, or the development of tumors associated with cell therapy. One potential risk is the development of an immune reaction or rejection of the infused cells, which can fail the treatment. Another risk is the potential for the infused cells to differentiate into unintended cell types, leading to unwanted side effects. Nevertheless, ongoing research suggests that cell-based treatments hold significant promise for addressing the underlying causes of RPL and RIF.

## 4 Stem cell-derived exosome therapy

Stem cell-derived exosome therapy is a fascinating and promising novel treatment approach for RPL and RIF. The therapeutic effects of stem cells, such as differentiation, cytokine release, and growth factor secretion, are mainly attributed to their paracrine activity rather than direct cellular integration [107]. Exosomes, small membrane-bound vesicles released by stem cells, contain signaling molecules like proteins, lipids, and nucleic acids [108]. These vesicles carry a variety of biological cargo, such as enzymes, heat shock proteins, microRNAs, mRNAs, tRNAs, DNA fragments, and cell surface proteins, including those involved in immune modulation and tissue repair. Some exosomes display specific surface proteins such as major histocompatibility complex (MHC) molecules, intercellular adhesion molecules, integrin, galectin, and collagen [[109], [110], [111]]. Isolated from stem cells, stem cell-derived exosomes have the potential to promote tissue regeneration and modulate the immune response, making them an attractive and intriguing option for treating conditions such as RPL and RIF. Exosomes trigger cellular processes such as proliferation, migration, angiogenesis, and apoptosis [107,[112], [113], [114]]. They also modulate gene expression, suppress the immune system, and promote embryo implantation by manipulating various signaling pathways [115]. The potential of exosome therapy in reproductive medicine is promising and exciting, as it opens up new possibilities for treatment and research in the field.

The rationale for exosome therapy in RPL and RIF lies in their ability to transfer signaling molecules to target cells, promoting tissue regeneration and modulating immune responses [107]. Exosomes derived from stem cells have demonstrated immunomodulatory properties, suppressing immune activity and reducing inflammation [108]. Exosomes do not elicit significant immunological reactions and can survive in an inflamed environment [109]. These properties are particularly relevant in RPL and RIF, where immune dysfunction often plays a role. Furthermore, exosomes can promote angiogenesis, which is crucial for maintaining a healthy uterine lining and supporting successful implantation [110]. During embryonic growth and development, the interactions between the embryo and the endometrium rely on releasing microvesicles/exosomes from placental components such as trophoblasts and luminal and granular endometrium [111,112]. Placenta-derived exosomes can suppress Tregs and the maternal immune system by secreting UL16 binding protein 1-5, MHCI, MHCII, and Fas ligand during pregnancy [[113], [114], [115]]. Endometrial-derived exosomes enhance vascular growth and ensure adequate blood flow by releasing miRNAs 21 and 126 [116].

Different types of exosomes can be used in therapy, including those derived from MSCs, ESCs, and iPSCs [[117], [118], [119]]. MSC-derived exosomes, in particular, have been extensively studied and have demonstrated immunomodulatory and regenerative properties. They promote angiogenesis and improve the thickness of the uterine lining. ESC-derived exosomes are under investigation, although their use raises ethical concerns due to embryo destruction. iPSC-derived exosomes represent a newer area of research, showing promise in preclinical studies [117,120]. The efficacy of exosome therapy for RPL and RIF is still being studied. However, its clinical application requires improvement due to the need for established cell culture conditions and optimal exosome production, isolation, and storage protocols. Ongoing challenges include determining the optimal therapeutic dose, administration schedule, and reliable potency assays to evaluate treatment efficacy. While limited clinical data are available, preclinical studies have observed promising results. However, no specific research currently investigates stem cell-derived exosomes in humans with recurrent miscarriages. Nevertheless, exosomes have demonstrated potential value in the treatment of pregnancy-related diseases. In a study by Yan-Jie Xiang, bone marrow MSCs were isolated from female mice, and exosomes were obtained from the cell culture medium through ultracentrifugation. Pregnant mice predisposed to abortion were treated with these exosomes [118]. The results showed improved pregnancy outcomes, with increased levels of IL-4 and IL-10 and reduced levels of TNF-α and IFN-γ at the maternal-fetal interface. The treatment modulated T cell function and macrophage activity, resulting in a decreased embryo resorption rate and demonstrating the therapeutic potential of ESC-derived exosomes in treating RPL [118]. Ebrahim et al. investigated the systemic administration of extracellular vesicles derived from human umbilical cord MSCs (UCMSCs-EVs) as a therapeutic agent for intrauterine adhesions (IUAs) caused by endometrial injury [121]. The study also explored the therapeutic impact of UCMSCs-EVs and estrogen separately and in combination in a rat model. The groups treated with UCMSCs-EVs alone or combined with estrogen showed significant decreases in inflammation, fibrosis, and vascularization compared to untreated rats with IUAs. The most important results were observed in animals receiving the combined therapy of UCMSCs-EVs and estrogen [121]. IUAs can be a cause of RIF or RPL [122]. Similarly, another study aimed to identify exosomes derived from adipose-derived MSCs (ADSC-exo) and explore their therapeutic potential in IUA rat models [123]. ADSC-exo promoted endometrial regeneration and collagen remodeling and enhanced the expression of specific proteins related to receptivity in the regenerated endometrium, ultimately restoring fertility [123].

While exosome therapy is considered safe, with a low risk of immune rejection due to its low immunogenicity, more research is needed to establish optimal dosing, timing, and storage protocols for clinical use. Nevertheless, the potential of exosome therapy in treating RPL and RIF is promising, particularly as an adjunct to current treatments.

## 5 Pharmacogenomics

Pharmacokinetics, introduced by Friedrich Vogel in 1959, encompasses how an individual's genetic makeup influences their response to medications [124,125]. Its research focuses on two main areas: identifying specific genes and gene products associated with various diseases to serve as targets for new drugs and identifying genes and allelic variants that affect individual responses to existing drugs [126]. Differences in drug response are multifactorial, resulting from the interaction of multiple genes with environmental factors [124]. This understanding led to the emergence of pharmacogenomics, enabled by high-throughput technology capable of studying entire genomes [124,127]. Pharmacogenomics, a biotechnological science integrating medicine, pharmacology, and genomics, aims to develop drug therapies tailored to individual genetic differences that lead to varied responses to treatment regimens [128,129]. Gene expression is influenced by various factors, including gene interactions, epigenetic changes, and environmental factors [124]. In multifactorial disorders, similar-looking diseases in patients may result from different genes, necessitating distinct drug therapies [124]. Personalized medicine utilizes a patient's genetic and environmental profile to predict the most effective drug therapy, reducing the risk of adverse effects [124]. However, the complexity of interactions involved in drug treatment presents challenges. Nevertheless, advancements have demonstrated that polymorphisms in genes encoding drug receptors, transporters, cell signaling pathways, and those involved in drug metabolism and disposition account for a significant portion of drug response variability [130].

For instance, adverse effects of drugs metabolized by genetically variable enzymes can be avoided by pre-testing patients for relevant genetic variants, administering the medication based on normal enzyme levels, or adjusting dosage according to metabolic state [124,130]. This pre-testing is typically limited to drugs with potentially higher toxicity [124,130]. While obtaining a complete genetic and environmental profile is impractical, genetic variation among populations can be a predictive tool, particularly regarding metabolizing enzymes that exhibit significant variation across people, influencing drug responses [124,130].

Pharmacogenomics holds great promise in the context of RPL and RIF. By utilizing genomic information, pharmacogenomics can personalize drug therapy, improving treatment outcomes and reducing the risk of adverse reactions [131]. Heparin, LMWH, aspirin, and progesterone can be personalized through pharmacogenomics in treating RPL and RIF [131].

Genetic variations in relevant genes impact the metabolism, efficacy, and response to these drugs, and pharmacogenomics can aid in identifying such variations and tailoring dosing to individuals [132,133]. Several pharmacogenetic tests are already used in clinical practice, such as the CYP2C19 test for clopidogrel antiplatelet therapy [134], and the CYP2C9 and VKCOR test for warfarin therapy as well as heparin and LMWH therapy [[135], [136], [137], [138]]. Clopidogrel antiplatelet therapy could be used for treating recurrent miscarriages in some cases [139]. Aspirin, a commonly used medication in treating RPL and RIF, can be personalized through pharmacogenomics. Recent studies have demonstrated that genetic variations in the COX-1 gene can impact the antiplatelet effect of aspirin, leading to variations in treatment outcomes [140].

Progesterone is frequently administered in RPL and RIF to support the luteal phase of the menstrual cycle and improve pregnancy outcomes [141]. However, determining progesterone therapy's optimal dosage and duration remains challenging, as these factors can vary among individuals. Genetic variations in the progesterone receptor gene have been identified as influential factors in the response to progesterone treatment. Notably, a study investigating the consequences of the PROGINS polymorphism, a relevant gene and protein aberration associated with the progesterone receptor, revealed a diminished response to progesterone [133].

By incorporating pharmacogenomics, these medications can be personalized to individual patients, maximizing their efficacy while minimizing the potential for adverse reactions. Pharmacogenomics helps optimize drug therapy and identifies individuals who may not respond to standard treatments, leading to the development of alternative strategies. It can reduce costs by minimizing trial-and-error dosing and reducing the risk of treatment failure. However, challenges remain in the field, including the need for genetic testing standardization of interpretation and large-scale clinical trials to evaluate the efficacy and safety of pharmacogenomics-guided drug therapy.

## 6 Treatment strategies

Traditionally, the treatment of RPL and RIF has involved a range of approaches, including hormonal therapies, immunomodulation agents, and surgical interventions [142].

***Hormonal Therapies****:* Hormonal therapies such as hCG have improved clinical pregnancy and live birth rates while reducing miscarriage. hCG administration is typically done intramuscularly. For a single dose, the usual dosage ranges from 5000 to 10,000 IU per injection. However, the dosage is significantly lower for daily or every other day administration during the luteal phase, typically around 1000 to 2000 IU per day [143]. Progesterone supplementation is effective in women with unexplained recurrent miscarriages. Progesterone is administered via intramuscular injection, vaginal suppositories, or oral tablets. Dosages range from 100 to 400 mg per day, initiated post-ovulation or embryo transfer, and continued until the end of the first trimester of pregnancy [144].

***Surgical Interventions:*** Surgical interventions such as hysteroscopy and laparoscopy have been used to correct anatomical abnormalities that may contribute to RPL and RIF. Hysteroscopy is a minimally invasive procedure involving the insertion of a hysteroscope to visualize and correct uterine abnormalities. Laparoscopy is a surgical procedure using small abdominal incisions to access and treat pelvic abnormalities like endometriosis or fibroids [142,145].

***Immunomodulatory Agents:*** Immunomodulatory approaches include anticoagulants, corticosteroids, intravenous immunoglobulin, and immunosuppressive medications to inhibit graft rejection, such as calcineurin inhibitors, recombinant cytokines, and cell therapy approaches [146]. Immune-modulating agents such as IVIG and corticosteroids have been used to suppress the immune response and prevent embryo rejection. Prednisone or prednisolone is typically initiated at 10–20 mg per day and tapered gradually. IVIG is administered intravenously at doses ranging from 0.4 to 2 g/kg body weight over several hours, with frequency tailored to patient response [142,144]. Aspirin and LMWH are commonly used in the treatment of RPL and RIF in women with antiphospholipid syndrome (APS), inherited thrombophilia, or idiopathic RPL. Aspirin and LMWH are widely prescribed and administered orally or subcutaneously, respectively [146,147].

While these traditional approaches can be practical for some couples, they may only be effective for some and may be associated with side effects and risks [11,148,149]. For example, the result of a study that treated women with RPL with prednisone and aspirin showed that the results were ineffective in promoting live births, and it increased the risk of prematurity [149]. The significant side effects of this therapy were hypertension and diabetes mellitus [149]. A systematic review by Achilli et al. on the role of immunotherapy in IVF and the management of RPL did not show a role for immunotherapy, such as IVIG, in improving the live birth rate in women undergoing IVF treatment or in the prevention of idiopathic RPL [11]. Therefore, novel approaches to managing RPL and RIF have emerged as promising strategies for improving treatment outcomes and minimizing the risks associated with traditional methods.

One of the main benefits of combining traditional treatments with precision and regenerative medicine approaches is the potential to personalize treatment based on the unique characteristics of each patient. This can be achieved using diagnostic gene profiles and other molecular information to identify underlying causes of RPL and RIF, which can be targeted with specific treatments [150,151]. Precision and regenerative medicine approaches can be combined with traditional therapies to offer couples experiencing RPL and RIF a range of personalized, targeted treatment options that may improve treatment outcomes and minimize the risks associated with conventional approaches, reducing the psychological stress associated with these conditions. They can offer several additional potential benefits, including long-term benefits with minimal risks, increasing the accuracy of disease models, characterizing individual genetic variations, and amplifying natural healing processes [150,152]. When combined with minimally invasive procedures, precision, and regenerative medicine approaches can allow patients to experience long-term benefits while avoiding unnecessary risks and potentially minimizing the financial pressure associated with these conditions [152]. Precision medicine approaches can improve the accuracy of disease models, which may enhance the efficacy of regenerative medicine strategies and improve translation to the clinic [152]. It can also be used to characterize individual genetic variations that may affect regenerative medicine outcomes [150]. Precision medicine involves tailoring treatment to an individual's specific genetic factors, which can reduce the risk of adverse reactions to medications and increase treatment efficacy [153]. Precision and regenerative medicine approaches may be combined with traditional treatments, such as immune-modulating agents, to suppress the immune response and prevent embryo rejection in women with RPL and RIF [150,151].

Limited information on specific examples of regenerative medicine approaches combined with traditional RPL and RIF treatments is available. However, by combining tissue engineering with conventional therapies such as surgery, clinicians can address anatomical abnormalities contributing to RPL and RIF and improve treatment outcomes. Tissue engineering involves using biomaterials and cells to create functional tissues that replace damaged or diseased tissues. Functional uterine tissue engineering requires highly specialized biomaterials with a natural extracellular microenvironment. Many efforts have been made to develop highly functional materials and tissue structures that may provide uterine tissue engineering constructs for reducing obstetric and gynecological complications [154]. Extracellular matrix (ECM)-derived materials have been used in uterine and ovarian tissue engineering to provide a natural microenvironment for cells to grow and differentiate. ECM-derived materials have improved endometrial stem cells' adhesion, proliferation, and differentiation. They may be used to address unfavorable maternal-fetal interface and improve fertility outcomes [[155], [156], [157]]. Bio-scaffold materials have been used as delivery strategies for therapeutics for endometrium regeneration. These materials can be loaded with stem cells or other therapeutic agents and implanted into the uterus to promote tissue repair and regeneration [158]. One report of a study on a 35-year-old female patient with RIF and a 39-year-old healthy husband showed that in the first two IVF cycles, despite the number of oocytes retrieved, estradiol (E2) levels, and normal uterus determined by hysteroscopy, the embryos failed to implant [159]. In these failed IVFs, the endometrium was <5 mm. In the third IVF, the first regenerative medicine intervention was suggested to the patient to address endometrial thickness. MSCs were applied and achieved a 7 mm lining. However, the embryo quality remained poor. In the fourth cycle, autologous PRP was isolated from venous blood by centrifugation to enhance ovarian function; the patient's response improved, and a euploid embryo developed. The baby was born after the embryo transfer and a normal pregnancy of 38 weeks [159] (Table 2).

**Table 2:** Treatment strategies for RPL and RIF.

Treatment Approach	Description	Clinical Implications
Hormonal Therapies	Use of hCG, progesterone, and other hormones to support pregnancy	Effective in improving clinical pregnancy and live birth rates
Surgical Interventions	Hysteroscopy laparoscopy to correct anatomical abnormalities contributing to RPL and RIF	Corrects structural issues that may affect fertility outcomes
Immunomodulatory Approaches	Anticoagulants, corticosteroids, IVIG, and immunosuppressive medications to suppress the immune response	Used to prevent immune-mediated embryo rejection, common in women with RPL and RIF
Aspirin and LMWH	Commonly used in RPL and RIF for antiphospholipid syndrome, inherited thrombophilia, or idiopathic RPL	Reduces the risk of thrombosis and improves blood flow to the uterus
Precision and Regenerative Medicine	Combining traditional treatments with personalized, targeted approaches based on individual genetic and molecular information	Offers personalized treatment options, minimizes risks, and improves outcomes

Abbreviations: hCG: Human Chorionic Gonadotropin, IVIG: Intravenous Immunoglobulin, LMWH: Low Molecular Weight Heparin, RPL: Recurrent Pregnancy Loss, RIF: Recurrent Implantation Failure.

While regenerative medicine approaches promise to improve treatment outcomes in RPL and RIF, much of the research in this area remains confined to the bench rather than the bedside. However, clinical translation is becoming apparent, and emerging technologies are expected to provide significant benefits in the long term [107,160,161]. Regenerative medicine offers new hope for improving pregnancy outcomes in RIF patients, mainly through the use of cell-based therapies and biologically active substances. PRP and granulocyte colony-stimulating factor (G-CSF) have been explored as potential treatments to enhance endometrial receptivity and support embryo implantation.

A recent randomized clinical trial compared the effects of intrauterine G-CSF and autologous PRP infusion in 200 patients with RIF [162]. The results demonstrated that patients who received PRP had significantly higher implantation, chemical pregnancy, clinical pregnancy, and ongoing pregnancy rates compared to those treated with G-CSF. Specifically, PRP resulted in a higher chemical pregnancy rate (P = 0.003) and ongoing pregnancy rate (P = 0.02), suggesting that PRP is more effective than G-CSF in enhancing pregnancy outcomes in RIF patients. This highlights the growing role of PRP in regenerative therapies aimed at improving endometrial receptivity and overall reproductive success [162].

## 7 Conclusion

In conclusion, RPL and RIF are devastating conditions for couples trying to conceive. Traditional treatment strategies for these conditions have limited success rates, and patients often experience significant emotional and financial burdens. However, precision and regenerative medicine approaches, such as diagnostic gene profiles, cell therapy, stem cell-derived exosome therapy, and pharmacogenomics, offer promising new avenues for treating RPL and RIF. Diagnostic gene profiles can identify genetic factors contributing to RPL and RIF, allowing personalized treatment approaches. Cell therapy and stem cell-derived exosome therapy offer regenerative methods that have shown promising results in clinical trials. Pharmacogenomics can help tailor drug treatments for individuals based on their genetic makeup, improving treatment outcomes. Dosage and mode of administration are critical in maximizing the efficacy of these therapies. hCG is typically administered intramuscularly, with dosages ranging from 5000 to 10,000 IU for single doses and 1000 to 2000 IU for daily or alternate-day administration during the luteal phase. Progesterone is given via intramuscular injection, vaginal suppositories, or oral tablets, with dosages ranging from 100 to 400 mg per day. Prednisone or prednisolone is usually started at 10–20 mg per day and tapered gradually. IVIG is administered at doses from 0.4 to 2 g/kg body weight, adjusted based on patient response. Aspirin and LMWH are prescribed orally or subcutaneously, respectively. MSC and exosome therapy typically involve the intrauterine delivery of cells. Exosomal protein therapy is timed during the luteal phase to optimize the window of implantation. There is limited evidence regarding the optimal doses of MSC and exosome therapy for intrauterine administration in RPL or RIF. PRP is also usually administered intrauterine in 1–5 mL volumes to improve endometrial receptivity. By refining these parameters, clinicians can optimize therapeutic outcomes and reduce potential risks.

Moreover, the combination of traditional treatment strategies with precision and regenerative medicine approaches may provide an even more significant benefit to patients. The potential impact of precision and regenerative medicine therapies on the lives of couples experiencing RPL and RIF cannot be overstated. By combining traditional treatments with precision and regenerative medicine approaches, clinicians can offer patients a range of potential benefits, including long-term benefits with minimal risks, increased accuracy in disease models, and the potential to cure disease. Future directions for precision and regenerative medicine in RPL and RIF include continued research into the mechanisms underlying these conditions and the development of new and innovative therapeutic approaches. These approaches may involve using novel cell types, gene editing technologies, and improved diagnostic tools.

## CRediT authorship contribution statement

**Kimia Motlagh Asghari:** Writing – review & editing, Writing – original draft, Project administration, Methodology, Investigation, Conceptualization. **Tannaz Novinbahador:** Writing – review & editing, Investigation. **Amir Mehdizadeh:** Writing – review & editing, Investigation. **Mohammadali Zolfaghari:** Writing – review & editing, Methodology. **Mehdi Yousefi:** Writing – review & editing, Supervision, Conceptualization.

## Declaration of competing interest

The authors declare no conflicts of interest in relation to this manuscript. No financial or non-financial interests have influenced the research presented in this review article. Additionally, the authors have no financial relationships with any organizations that might have an interest in the submitted work within the past three years.
